# The Perceived Threat in Adults with Leukemia Undergoing Hematopoietic Stem Cell Transplantation

**DOI:** 10.5812/nms.11243

**Published:** 2013-06-27

**Authors:** Zahra Farsi, Nahid Dehghan Nayeri, Reza Negarandeh

**Affiliations:** 1 Department of Medical Surgical, Faculty of Nursing, AJA University of Medical Sciences, Tehran, IR Iran; 2 Department of Management, Faculty of Nursing and Midwifery, Tehran University of Medical Sciences, Tehran, IR Iran

**Keywords:** Cancer, Fear, Grounded theory, Leukemia, Hematopoietic stem cell transplantation, Threat

## Abstract

**Background:**

Leukemia and hematopoietic stem cell transplantation (HSCT) create physical, psychological, social, and spiritual distresses in patients. Understanding this threatening situation in adults with leukemia undergoing HSCT will assist health care professionals in providing holistic care to the patients.

**Objectives:**

The aim of the present study was exploring the perceived threat in adults with leukemia undergoing HSCT.

**Patients and Methods:**

This article is part of a longitudinal qualitative study which used the grounded theory approach and was conducted in 2009-2011. Ten adults with acute leukemia scheduled for HSCT were recruited from the Hematology–Oncology Research Center and Stem Cell Transplantation, Shariati Hospital in Tehran, Iran. A series of pre-transplant and post-transplant in-depth interviews were held in the hospital’s HSCT wards. Totally, 18 interviews were conducted. Three written narratives were also obtained from the participants. The Corbin and Strauss approach was used to analyze the data.

**Results:**

Perceived threat was one of the main categories that emerged from the data. This category included four subcategories, "inattention to the signs and symptoms", "doubt and anxiety", "perception of danger and time limitation" and "change of life conditions", which occurred in linear progression over time.

**Conclusion:**

Suffering from leukemia and experiencing HSCT are events that are uniquely perceived by patients. This threatening situation can significantly effect perception of patients and cause temporary or permanent alterations in patients' lives. Health care professionals can help these patients by deeper understanding of their experiences and effective interventions.

## 1. Background

Cancer diagnosis is always accompanied with stress and it can create constant or temporary physical and psychological changes in patients (1). Complications of cancer may lead to hopelessness, unbearable pains, fear and even death (2). One of the most frequent cancers is leukemia which had appeared in 12.5 individuals among every 100,000 people in 2011 (3). Today, one of the most important and advanced medical achievements is the development of transplantation which has been accepted worldwide during a relatively short period as a modern and competitive method to cure many patients (4). Hematopoietic stem cell transplantation (HSCT) is a treatment method for some hematological malignancies such as leukemia (3, 5-7). The method requires intravenous infusion of hematopoietic stem cells (bone marrow, peripheral blood or umbilical blood stem cell) in order to reorganize bone marrow function in patient whose bone marrow have been injured or weakened (8, 9). During the recent two decades, HSCT has had a fast growth. More than 25,000 patients in Europe are being cured through this method annually and globally this number increases to 100,000 cases (10). It has been estimated that in the United States, there are 274930 patients either suffering from leukemia or have recovered from this disease, many of whom have undergone HSCT (3). 

Bone marrow transplantation (BMT), started in Iran since March 1991 (8). Currently four HSCT centers are active in the country but the need for other centers is felt, considering the deployment of noncontagious diseases specially cancers. Currently in Shariati Hospital’s Hematology - Oncology Research Center and Stem Cell Transplantation (HORCSCT), there are more than 60 standard transplant units (8), and more than 3000 HSCT have been performed in this hospital (11).

Although success of HSCT has increased as a result of supporting care and better perception of immunological changes of transplantation (9), this procedure can have physical and emotional stresses due to the toxicity of treatment regiments (combination of high dose chemotherapy with or without radiotherapy), transplantation process and its longtime consequences (7). This method effects different systems of the body and is accompanied by acute and chronic complications. Different reports have pointed out various physical, psychological and social problems of these patients in relation to transplantation (5, 12-14), such as chemo radiotherapy effects, infections after transplantation, recurrence of primary disease, hypothyroidism, secondary cancers, cataract, infertility (12), graft versus host disease (5, 12, 14), sexual disorders, early menopause, isolation, distress, furiousness and anger, fear of recurrence (5, 8, 13), post traumatic stress reactions, cognitive disorders, occupational disability, low self-confidence, problem in adjustment and disorder of social roles, feeling loss of self control (5, 8) and fear of death (3, 5, 8).

Koenigsmann et al. in a qualitative research have studied the perceptions of adults, suffering from acute leukemia, about their illness, lay theories and coping strategies one week after diagnosis. Experience of cancer resulted in feelings such as fear, helplessness and effort of normalization (15). Stephens in a phenomenological study investigated lived experience of patients who had undergone autologous HSCT. Findings of her study indicated that experience of transplantation could influence a person's life for an undetermined period of time. Experienced changes might be permanent and after transplantation the person wouldn’t be the same regarding their emotional, psychological and physical point of view (7). Assessment and perception of patient’s problems confronting stresses related to the disease and transplantation is very important for the nurses and other healthcare staff; i.e. knowledge about the patients' crisis before and after transplantation can help the healthcare professionals in development of supporting interventions, submission of information and giving appropriate advices. Therefore, considering the importance of understanding these patients' perceptions about the threats and its important role in promotion of holistic nursing care, necessity to pay attention to this phenomenon is deeply felt in Iran, while there are no studies dealing with this matter in Iran. Qualitative researches can have an effective role in clearing ambiguities in this regard.

## 2. Objective

The researcher's purpose was to explore the perceived threat in adults with leukemia undergoing hematopoietic stem cell.

## 3. Patients and Methods

This article was part of a qualitative research which was done based on the methodology of grounded theory described by Corbin and Strauss (16) from 2009 to 2011. It is essential to mention that in this paper only one of the major categories was reported and some other categories have been reported elsewhere (1, 11). The participants were recruited based on following inclusion criteria: candidate HSCT patients with diagnosis of leukemia, ability to speak and comprehend the Persian language, 18 years old and older, convenient psychological and physical conditions for participation in the research, and preference to participate.

The participants were recruited from HORCSCT in Tehran as a referral center from the entire country. The first participant was selected through purposeful sampling. By progress, theoretical sampling replaced purposeful sampling and continued until theoretical saturation occurred. . Interview with a patient who had realized recurrence of his disease two days before transplantation, a patient who had second experience of transplantation and a patient during the transplantation were examples of theoretical sampling.

Open in-depth interviews were the main method for collection of the data. Total interviews were conducted by one of the researchers (Z.F.). Interviews started with questions like: "Please speak about the moment when the disease signs appeared to you. What happened when you understood you have this disease? How did you feel at the moment of diagnosis of the disease? What does this disease mean for you?" The participants were interviewed once to three times during a 7-month period (for each patient). Collection of data in several different terms enabled researchers to capture the patients' experiences with threatening situation .In addition, interviews allowed the researcher to attain a deeper perception of the phenomenon under study. In fact, repetition of interviews at different times led to the appearance of categories and concepts which hadn’t appeared by doing one interview with each participant. The interviews were done in the patients' private inpatient room or, if post discharge, in a private outpatient room in HORCSCT. All follow-up interviews were done based on previous coordination with the patients. The patients were isolated during hospitalization, but they were allowed to visit their family during particular hours from the back of their rooms' window and had verbal communication through the telephone with them. Of course by permission of the transplantation units' managers the researcher could interview with patients face to face in an isolated room observing the isolation principles (using mask, gown, gloves etc.). Each interview lasted between 40 to 120 minutes depending on the condition and participants' cooperation. Primary interviews, which were done before the transplantation, were longer in duration. By coordination with and obtaining permission from the patients, all of interviews were recorded by a digital voice recorder. Simultaneously, by reviewing the field notes, which had been written by the researcher immediately after the interview, the interviews were transcribed verbatim. During the research period, 18 interviews were done with 10 patients and 3 written narratives were collected. The main criterion of researcher regarding the number of interviews was usage of key informants, collected data, emerging categories and theory and attaining to theoretical saturation. Field notes, unstructured observations, and written narratives were the other methods for collecting data. The researcher collected, coded and analyzed the data simultaneously. Open, axial and selective coding, according to Corbin and Strauss paradigm (16), allowed a structured data analysis process. For instance, in the open coding process, quota included the following: "the patients on the second floor usually have a relationship with each other. For example, they ask about each other’s problems? What doctors did and didn't tell them? Unconsciously this fear existed for everybody." first level codes like "relationship with the patients", "getting information" and "fear of transplantation" emerged. This act was done for all interviews and initial codes were placed in the list of open coding.

Also, the method of constant comparison at all stages of analysis was applied, and similarities and differences between primary codes were determined. Similar codes placed in the same category and were conceptualized. The codes were reviewed in a rounding movement regularly and were revised if necessary. The interviews with each patient were reviewed individually; they were also compared with primary stages' interviews so that the process of changes in perceptions and experience of the patients could be specified. In addition, all interviews were compared with each other. The researcher in the axial coding process reviewed the existing meaning of the data. For instance:

“When I knew I had to undergo transplantation a second time (condition), I cried hard (coping strategy) because transplantation was too hard to bear. I was quite scared (outcome).”

Conditions, strategies and outcomes were extracted and the relationship between categories and central category was determined through selective and axial coding process. Comparison of data, asking questions, writing the story line, drawing diagrams and reviewing memos were the applied through this stage.

Guba and Lincoln have claimed credibility, dependability, fittingness and confirmability as the criterions of trustworthiness in qualitative researches (16). Making an appropriate relationship with the participants, doing serial interviews, prolonged engagement with the phenomenon under study (more than two years), checking the findings with participants, reviewing of interviews and coding process by peers, using the various methods for collecting data, limited review of the literatures, precise recording and reporting of all stages of the research were the methods which were applied for increasing the trustworthiness of this research. Also, the researcher's previous the experiences in caring for patients undergoing HSCT and co-working with nurses who have looked after them helped her in better understanding of the patients experiences.

This research was confirmed by the Ethical Committee in the Research Department of Tehran University of Medical Sciences and researchers promised to observe the stated ethical considerations in the Declaration of Helsinki (17). Ethical basics such as getting the informed consent, protecting anonymity, confidentiality of the information, the right of refuse to participate in the research for the participants, respecting the rights of the authors and receiving approval from the respected managers were considered in this research.

## 4. Results

The participants of this research were 10 adults suffering from leukemia undergoing HSCT. Patients' ages ranged between 18 - 48 years old (29.3 ± 10.1). Five patients were male and 7 patients were married. Eight patients had diplomas and higher degrees. Six patients were suffering from AML and four patients were suffering ALL. On average, the duration between diagnosis of the disease and transplantation was 6 months. Except for one patient who was experiencing his second transplantation, the others were being transplanted for the first time. All of the participants were Muslims. Only one of the patients was resident of Tehran and only one patient was transplanted autologous.

Suffering from cancer is a painful experience which makes the person's believes and perceptions challenging in coping with this phenomenon. In fact, the person's perception depends on the meanings associated with the phenomenon and strongly affect on his/her coping strategies for controlling and managing the situation which is "experiencing of cancer" in this study. Perceived threat was the main concept emerged from the data, which was defined as “the person's perception from feeling of danger of health status deviation, facing disease and probable consequences of disease and its treatment (like disability, injury, death).” The perceived threat was formed in four stages of "inattention to the signs and symptoms", " anxiety and doubt ", "perception of danger and time limitation" and "change of life conditions" since the time of appearing signs and symptoms of disease till after the transplantation in patients.

### 4. 1. Inattention to the signs and symptoms

Since the patients didn’t expect to be faced with a serious disease, they didn’t pay as much attention to the signs and symptoms of the disease before diagnosis and related them to their prior conditions and previous experiences. One of the female participants said:

“I tailored too much so I got knee pain. I thought it might be because of tailoring. I went to an orthopedist. He didn’t recognize it and told me that my tendon has been stretched and he just prescribed me drugs. I didn’t pay attention to my symptoms and I remained ignorant of my disease for too long” (P4).

### 4. 2. Anxiety and doubt

With time and progression of signs and symptoms, anxiety and doubt about the disease was created in the patients; in a way that they became more sensitive and sought for the reason and realized the danger which had threaten their life. A 23-year-old participant said:

"I had a nose bleeding then my gums bled. For a long time, when I woke up at nights, I saw my gums bloody. I changed my toothbrush, but it was not useful. One day, my gums hemorrhaged, I realized that the reason is not the toothbrush. I couldn’t walk much, I got tired soon. I wondered why I have become like this, I had become pale. My platelet was reduced. Finally I referred to a physician" (P3).

### 4. 3. Danger perception and time limitation

At the time of diagnosis, the patients were shocked and didn’t believe it; most of them were talking with choky voice or crying while they were speaking about it. With diagnosis of the disease and awareness about the cancer, the patients gradually understood the dangers, which threat their life and cancer became a source for them. A 25-year-old woman who did not have as much information about her disease till a few days before transplantation said:

"When I heard (I had cancer), I became daunted. The doctor told me if you do transplantation, it has a 46% success rate… In short, he really made me scared "(P4).

At this stage, the participants were looking for the reason why they got cancer and they related it to different factors. Attribution, denial and avoiding, evacuation of excitements, facing reality, accepting the condition, organizing treatment, looking for social support and leaning toward religion were the most applied coping strategies by the patients at this stage.

"Whenever I am sad, I cry, this way, I'll become relaxed. I thought the reason for it was God’s will, I accept it".

The patients, who were being prepared for transplantation by passing the treatment stages and thought of transplantation as their only way of survival, were afraid of losing time and failure in transplantation. The participants believed that the word "cancer" creates fear in many people. One patient knew cancer as the worst disease and it caused them to perceive danger. In the primary stages, they thought more about complicated treatments or even refractory of their disease. Most of the patients realized their serious problem after awareness of the necessity for transplantation. Some of them knew transplantation as a difficult surgery and most knew it as an inevitable cure. A participant said:

"Well, transplantation wasn’t an evitable cure to make me completely happy, but I was a little happy because I pass some stages of treatment; on the other hand I was realist due to failure in some stages of transplantation " (P5).

All participants had felt the limitation of time. A middle-aged man said:

"The doctor did really emphasize that I should do the transplantation approximately within two or three months. I went to hospital to book. However I insisted on having the earliest time, they told me to go and come back on the 3^rd^ of December. I said to the laboratory doctor: please prescribe urgently; my doctor has emphasized me to do it as soon as possible "(P2).

### 4.4. Change in conditions of life

Change in conditions of life was considered as another threat for patients. Suffering from cancer and experiencing different treatment stages had led to a change in normal life of participants, which annoyed them; as a result, they had the desire of returning to normal life. The data indicated that suffering from leukemia and its cure such as HSCT is an exclusive experience, which has led to create wonderful changes in the patients and these changes might have been constant. In fact, the transplanted patient wasn’t the same person from the physical, psychological, emotional, spiritual and social point of view, in other words, he/she was reborn. In this new experience, change in normal life was considered as one of the main issues of the patients. "Change of physical function", "threat to important individual objectives", and "social isolation" were placed under this concept.

Leaning toward religion, looking for social support, behavior modification, reflection and patience were the most important coping strategies used by the patients at this stage.

#### 4.4. 1. Change in physical function

The changes, which are created in physical function of the patients by cancer and its treatment, were one of the causes of their anxiety. As the signs and symptoms of the disease and complications of treatment were intensified, the amount of fear and stress increased in the patients. They knew intensifying of these changes as one of the signs of approaching death; and during these times, the amount of their perceived threat increased. As a matter of fact, disease concept had a determining role in the patients' perception of the situation. The patients were annoyed by appeared intensive complications of treatment, obligations of intensive self-care, nutrition limitation, consumption of too much drug, and they were worried that this status would continue after leaving the hospital. Drug consumption, appearance of gastrointestinal complications, changing food-serving time, obligation of being hospitalized and loneliness in the isolation room resulted in the reduction of appetite and consequently weight loss. Some patients preferred home food to hospital's food and patients who had enough facilities and appropriate conditions did so. A patient who was experiencing her second transplantation said:

"I vomited after eating the pills. I hate vomiting. In a moment, I saw my whole bed and dress dirty. After that I realized that I couldn’t even control myself (fecal incontinence). I like to have 3 to 4 meals so that I stop feeling weaken. When I go to the toilet, my anus really burns and really aches. “(P10)

A 26-year-old man mentioned:

"My throat was scratched and it ached, I had diarrhea. I had become weak physically and I lost weigh about 8 kg" (P1).

#### 4.4. 2. Threat to important individual objectives

Most patients believed that generally before diagnosis of the disease, everything was going well, but the disease caused corruption of everything. Since the patients saw the approach of death and they desired to live, they had to adopt strategies in line with deliverance from death. Because of that, they had to renounce the important goals of their life. The feeling of leaving their loved ones behind, corruption of future, instability in occupational successes, to be deferred from education, losing their favorite things, being forced to start over, wasting time and financial loss were among the emerged codes from the data which were considered as threats to the important goals of their life. One of the participants, who was a student and had been diagnosed with the disease said:

"I feel the disease has corrupted my future life, I was deferred from education, and I was constructing my future very well and was a clever boy. I saved my money, I was a powerful and special person but the disease left nothing for me; well, it's hard to start over" (P3).

Two of participants who had planned to have a child for a long time, after they were informed about their cancer; they had to abort to save their life which was a very painful experience for them:

"Of course this is hard for a mother. Because the doctors in the hospital said: they must announce the abortion to restart my medical treatment. I was very upset, I even requested to protect the fetus's life till eight- month-old to be born through cesarean section, but they didn’t agree. They said the disease is emergent and we can't postpone the treatment for protection of the child. So I accept to abort to save my life" (P9).

#### 4.4. 3. Social isolation

Due to the cancer and start of the treatment, the patients were kept aloof from social life. In fact, because of this restriction, some of their previous roles in the society were threaten. The patients suffered from being forced to stay at home and to tolerate social isolation in interruptions between chemotherapies and after the transplantations. A 23-year-old man said:

"I have stayed at home for six months; there was nobody to ask me to work or to study any more. I have been far apart from social life for six months" (P3).

The patients preferred to do their previous favorite activities the same as before and to enjoy life but it was impossible and they had to avoid attending some environments.

The patients must be isolated in a room for doing chemotherapy and transplantation and the conditions of isolation for transplantation were more intensive than chemotherapy's conditions. Toleration of isolation conditions for a long time with a fatal and serious disease was very oppressive for them. Feeling of being in prison, and negation of freedom, fear of disconnection from the outside of isolation room, lack of some required facilities such as internet access and direct phone line were the issues which had made passing time difficult for some patients. Although primary facilities had been prepared in the isolation room and there was possibility of having conversations with families and friends through the telephone, cell phone or iphone during visit times from behind of the glass window, most patients were dissatisfied due to the absence of their family members and a companion in conversation. Some believed that no attention is paid to the excusive needs of the patients; so they compared themselves with prisoners. They described prison as the worst torture for human and saw themselves imprisoned in the place for torturing:

"We must be confined to bed for at least for one month. One month is not a short time to be in an environment of isolation. You know, especially loneliness is the worst torture for a prisoner, negation of freedom is really hard and to have this disease is more difficult" (P1).

This group of patients believed that fulfilling the particular and exclusive needs of the patients can be an important factor to reinforce the sprit and a strong facilitator for coming to terms with the disease and not paying attention to this annoyed them:

"For instance, they should ask the patients what they need …for example, for a person who needs internet, they should prepare it." (P1).

Loneliness was an important factor leading to the attack of negative thoughts. To be skein, depression and even hallucination were some of the items to which the patients pointed. For example, a woman talked about a hallucination incidence at the night before transplantation:

"I told the nurse that I am going crazy, I consecutively think my child has slept next to me, I got up to put a blanket over her. I realized that my child isn’t here and I am in the hospital "(P4).

Generally the patients were involved in emotional restriction and social isolation. It seems they had been imprisoned like "birds in cage" and they were restricted to fly. The longer the hospitalization term, the more difficult was the toleration of the conditions; the patients had developed different coping strategies.

## 5. Discussion

The purpose of this study was to explore the perceived threat of patients with leukemia undergoing HSCT. The perceived threat was the main extracted conception of data, which effected the patients' reaction towards the new condition. Diagnosis of cancer always creates stress (18) and can lead to serious psychological problems in a person (2). Although some diseases such as leukemia and their treatments are a threat for the patient's life, there are more psychological reactions depending on the patient's perception of the disease than the actual nature of the disease itself (19). In the current research, findings indicated that individual interpretations and perception of situation are strong determinants for coping with the threatening situation of life. Coping is the result of the individual perception of threat (20). In this research, the participants, before the disease diagnosis, related its signs and symptoms to their background conditions and previous experiences. Koenigsmann et al. also reported that adults suffering from acute leukemia relate the disease signs to other diseases before diagnosis (15).

In the current study, by passing the time and intensifying signs and symptoms and attendants reaction, the patients would become involved in "anxiety and doubt". In this kind of situation, most patients talked directly and sometimes indirectly about their life being under threat. Similar results have been reported in other researches (7, 15).

A Pervious study has indicated that cancer diagnosis has always been associated with stress and has the capability to create permanent changes in individual’s life (18).

In this study, the participants were shocked and had unbelief at the stage of disease diagnosis. Rahemi writes according to Sadeghi by hearing the word "cancer", most patients and their families are unwillingly involved in shocks (21). In this research, participants gradually realized the seriousness of their problem according to the existing evidence and gained information. Similar findings have also been reported in the research by Koenigsmann et al. These researchers note that the patients consider the disease not only as a threat for their body but for the entire person. In their study, the patients pondered severely in primary stages of the disease and attempted to estimate the seriousness of their situation based on the conditions (15).

In the current study, patients in the diagnosis stage had severe mental engagement with the disease and the reason behind their disease. By passing the time, the effect of the disease on their normal life changed their mental issues. The changes and restrictions on their normal life were one of the most important mental issues especially between chemotherapies and after transplantation. This finding is in line with the results of Rahemi's research. She reported that patients suffering from cancer don’t think about the nature of the disease too much, they care more about its effect on their life, the changes regarding continuation of their activities, job, their family and losing hope for the future (21).

The patients were annoyed by the created changes and restrictions after diagnosis of the disease. Change in physical function, threat to important individual's goals and social isolation were the most important items, which the patients were pointing out. Most patients pointed out that they never expected to confront such a threatening disease. In this regard, Miedema et al. also reported that the patients were busy with various activities and described themselves as an invincible person, they enjoyed their life, and this idea never came in their mind that they might develop cancer, most of them felt that they were stuck and they were suddenly filled with the feeling of vulnerability (18).

As the signs and symptoms of the disease were intensified, stress and fear in the patients were increased and they considered it as the approach of death and consequently the amount of perceived threat were increased. Also, it was noted that the perception of threat has a close relationship with the general meaning by which patients define the disease. Turk et al. have reported that the patients suffering from chronic pain of cancer in comparison with the ones suffering from the chronic pain related to other diseases had greater physical disability and restriction in their activities. More intensive disability and movement limitation of the patients was probably derived from the meaning of pain for these patients: having intensifying pain is the sign of disease progression, losing physical strength and approaching death (22). Threats to important individual objectives were one of the other extracted conceptions of data. The patients had to ignore many important goals of their life for being alive and survive death. Wu et al. have reported that adolescents suffering from cancer, after being informed about the disease, realized that they should sacrifice some of their previous goals for protecting their health and life. These researchers have called this lived experience "reorganization of thoughts" which was taken into account as one of the major stages of reconstruction of hope in the patients (22).

Social isolation was also one of the other dimensions of change in life conditions. The patients had to avoid others to prevent treatment complications and this led to create many limitations for them. Through these limitations, some of their previous roles in the society were threatened. In the isolation room, the patients saw themselves as a prisoner and they had to keep aloof of social life between chemotherapies and after transplantation. In Stephen's research, leaving the hospital after transplantation and confronting infections and tiredness at home were considered as feeling of being isolated. She has mentioned that life after transplantation involves isolation, physically and emotionally and confronting death will increase the feeling of isolation further (7).

The health-care staff should notice to give enough opportunity for the patients in order for them to express their feeling and stating their thoughts and to support them spiritually.

One of the limitations of this research was the patients' physical situation. According to the point that the patients undergo high-dose chemotherapy before transplantation, most of them suffered from gastrointestinal complications such as diarrhea and they needed to go to the toilet repeatedly. Sometimes during the interviews, the researchers had to interrupt the interview for minutes to fulfill the physiological needs of the patients. However, the researcher did her best to make the in-depth interview effective while she protected the patients' right and privacy.

In general, the findings of this research emphasize on having deeper perception of the patients' experiences like their perception of disease and transplantation. The professional health-care staff specially the nurses, can help care for the patient by deeper understanding of the experiences and perceptions of this group of patients and implementing appropriate interventions such as knowing the factors causing fear and stress in patients and adjusting them; knowing exclusive needs of the patients and trying to fulfill them; informing properly and on time; paying more attention to the spiritual needs of the patients; preparing necessary facilities in line with social support; understanding the patients and having a better relationship with them and giving good advise, paying more attention to the concept of family, culture and religion in providing care to the patients and preparing enough facilities. Eventually, doing similar researches in other societies with different cultures and religions is suggested.
